# Impacts of *Bacillus amyloliquefaciens* and *Trichoderma* spp. on Pac Choi (*Brassica rapa* var. chinensis) grown in different hydroponic systems

**DOI:** 10.3389/fpls.2024.1438038

**Published:** 2024-09-23

**Authors:** Gretchen Plocek, Dario Rueda Kunz, Catherine Simpson

**Affiliations:** Urban Horticulture and Sustainability Laboratory, Texas Tech University, Plant and Soil Science, Lubbock, TX, United States

**Keywords:** biofertilizer, beneficial microorganisms, deep-water hydroponics, nutrient film technique, microorganism-root interactions

## Abstract

Soilless production systems (i.e hydroponics, aeroponics, aquaponics) have become commonplace in urban settings and controlled environments. They are efficient nutrient recyclers, space savers, and water conservers. However, they lack high levels of biological richness in the root microbiome when compared to soil production systems, which may affect plant health and nutrient uptake. To address this issue and incorporate more sustainable practices, beneficial microorganisms (i.e. *Trichoderma* spp., *Bacillus* sp.) can be added in the form of biofertilizers. However, many factors affect impacts of microorganisms and their interactions with plants. In this experiment, Black Summer Pac Choi (*Brassica rapa* var. Chinensis) was grown for two trials in a Deep-Water system (DWS) or a Nutrient Film Technique system (NFT) with commercial biofertilizers containing *Trichoderma* spp., *Bacillus amyloliquefaciens*, a combination of both, and a control. Plant physiology, nutrient composition, and nutrient uptake efficiency (NUE) were generally negatively affected by *Trichoderma* spp. both growing systems, indicating that *Trichoderma* may not be recommended for hydroponic production. However, *Bacillus amyloliquefaciens* showed promise as an effective biofertilizer in the NFT systems and had a positive influence on NUE in DWS.

## 1 Introduction

Various forms of hydroponics in urban food production systems are set to increase in the near future to accommodate 55-68% of the global population living in cities (Rajaseger et al., 2023). Hydroponics are often used in greenhouses and urban farms as they allow for an environment that can easily be controlled and optimized for high-value crops (i.e., nutrient additions, water conservation, biofortification, temperature) (Sambo et al., 2019). Hydroponic systems are widely used to produce leafy green vegetables that are of high value. The vast majority of these being leafy greens (Walters et al., 2020). However, most hydroponic setups lack natural levels of biodiversity in the rhizosphere. This lack of biodiversity has been shown to decrease the efficiency of nutrient uptake by plants, increase disease susceptibility, and decrease bioavailable nutrients (Lin et al., 2021; Setiawati et al., 2023; Kumar et al., 2022; Chaudhary et al., 2022). Biofertilizers can be added to hydroponic systems’ nutrient reservoirs and rhizospheres to remedy the lack of biodiversity.

Biofertilizers are generally defined as mixtures of microorganisms (e.g., fungi, bacteria) added to the rooting environment to promote a crop’s growth, plant health, and nutrient uptake (Daniel et al., 2022; Mohammadi and Sohrabi, 2012; Zhao et al., 2024). Due to the positive associations that can be formed, these can also be termed beneficial microorganisms. Various microorganisms, such as *Bacillus* spp., *Trichoderma* spp., *Azospirillum* spp., and *Pseudomonas* spp., can be used as biofertilizers (Reed and Glick, 2023). Inoculations can be made through lab-grown populations or more easily accessible commercial products. Often, mixtures are added to the rhizosphere of crops grown in soil to achieve the desired effects of increased growth, biomass, and harvestable material (Dasgan et al., 2023). However, these desired effects may not be as easily reached in hydroponics due to rhizosphere environmental differences between soil-based and soilless production systems and compatibility challenges with various crops.

Biofertilizers used in hydroponic systems have varied in composition and ranged from microalgae (*Chlorella vulgaris)*, arbuscular mycorrhizal fungi (AMF), plant growth promoting rhizobacteria (PGPR), *Bacillus* spp., *Pseudomonas fluorescens*, *Glomus* spp., *Gigaspora margarita*, *Azobacter* sp., *Azospirillum*, sp., and many others have been explored (Dasgan et al., 2023; Kumar et al., 2022; Dasgan et al., 2022; Singh et al., 2023; Chaudhary et al., 2022). These systems have ranged from nutrient film technique (NFT), to floating raft, dutch bucket, deep water, and other systems (Dasgan et al., 2023; Ikiz et al., 2024; McClintic et al., 2024; Zhang et al., 2023). In these systems, researchers found that biofertilizers increased leaf physiological characteristics, plant yield, nutrient uptake, and higher phenol and flavonoid contents (Zhang et al., 2023; Ikiz et al., 2024; Dasgan et al., 2023). However, McClintic et al. (2024) found that the inoculation of *Azopirillum* and *Rhizophagus* sp. had limited effects on nutrient uptake and growth in tomatoes and lettuce. This variation in effects could indicate factors such as environment, storage, pH, incompatibility, nutrient competition, form, that may influence performance of biofertilizers (Karapetyan, 2023). Biofertilizers can be affected by storage, humidity, temperatures, or formulation issues that may be detrimental to live microorganisms (Karapetyan, 2023; Jain, 2019; Pirttilä et al., 2021). Hydroponic conditions may further complicate the relationship between plants and biofertilizers because of the nature of the nutrient and water solution. While liquid formulations may be preferable because of solubility and ability to apply via foliar spray, or interactions may be more efficient with root inoculation (Sarbani and Yahaya, 2022). However, several studies have shown positive impacts of foliar sprays of different biofertilizers, including increased enzyme activity, metabolites, nutrient concentration, mitigated effects of salinity, and increased biomass (Razmjooei et al., 2022; Vela Coyotl et al., 2018; Guimarâes et al., 2020). Yet one of the primary reasons for utilizing biofertilizers is to facilitate nutrient uptake or efficiency of uptake for organic nutrients. Current research has proven the efficacy of biofertilizers to facilitate uptake of organic nutrients, and sometimes inorganic nutrients is inconsistent (McClintic et al., 2024; Priadi and Nuro, 2017). This may be a function of different growing conditions, hydroponic conditions, plant species, or fertilizer, however, this must be studied further to determine exact causes and correlations between biofertilizers and their performance. Moreover, biofertilizer behavior can change based on environmental stress (Backer et al., 2018; Malusà et al., 2016). Vast environmental differences exist between soil-based production and soilless production systems, which may be a source of stress for populations of microorganisms. Some of these differences include particle porosity (which provides contact points for colonization) (Girvan et al., 2003; Vieira et al., 2020), available oxygen, leaching, and nutrient availability (i.e., affects symbiosis behavior) (Malusà et al., 2016). Yet biofertilizers have shown promise in mitigating negative plant responses to stressors (Chaudhary et al., 2022). Various biofertilizers have inhibited pathogen growth, regulated signaling pathways, increased gene expression, improved plant growth, proline synthesis, secondary metabolites, among many other responses (Chaudhary et al., 2022). For example, Guimarâes et al. (2020) found that biofertilizer mitigated some of the negative effects of salt stress in hydroponic lettuce. Further, Yasmin et al. (2020) found that priming soybean (*Glycine max*) seeds with *Pseudomonas pseudoalcaligenes* improved salt tolerance by increasing defense responses as compared to *Bacillus subtilis*. This contrasts with findings by Mokabel et al. (2022), who found that *Bacillus subtilis* had enhanced pigment concentrations, upregulated defense compounds, and enzymes compared to uninoculated eggplants *(Solanum melongena*). This further demonstrates the wide variability between crops, even when treated with similar stressors and the same biofertilizer.

Biofertilizers also have different compatibilities with desired crops. For example, mung beans (*Vigna radiata* L.) have been shown to react positively with *Rhizobium* spp., *Trichoderma viride*, and *Pseudomonas putida*, while rice nearly doubled in nitrogen (N), potassium (K), and phosphorus (P) concentrations after being given only *Rhizobium* isolates (Gangwar et al., 2013; Khanam et al., 2022). In addition, *Trichoderma* spp. has been documented to positively affect crops across taxonomic families and species (Kubheka and Ziena, 2022). For example, Chinese cabbage with different *Trichoderma* fungi applied via irrigation resulted in a significant increase in cabbage yield (by 37%) and enhanced enzyme activity in the soil (Ji et al., 2020). *Trichoderma azevedoi* also increased chlorophyll content in lettuce while decreasing white mold severity (Silva et al., 2021). Due to this pattern, *Trichoderma* was chosen as a representative commercial biofertilizer.

To further examine specific biofertilizers, Bacillus and Trichoderma, the following studies have been reviewed. For the case of *Bacillus*, maize given *Bacillus pumilus* resulted in a 30% increase in ear yield, but no significant results were documented when given *Pseudomonas moraviensis* (Kuan et al., 2016; Lin et al., 2018). In addition, various strains of *Bacillus subtilis*, *Bacillus megaterium*, and *Bacillus pumilus* have resulted in rate of growth increases, stress tolerances, and nutrient metabolites in lettuce and tomatoes (Radhakrishnan et al., 2017). Furthermore, *Bacillus subtilis* and *Bacillus cereus* positively impacted Chinese cabbage growth and nutrients (Kang et al., 2019; Ku et al., 2018). Due to this pattern, *Bacillus* was chosen as a representative commercial biofertilizer. *Bacillus* strains have not always resulted in positive impacts on plant growth, with a study by Moncada et al. (2021), showing reduced nitrate in basil leaves. Although few of these studies exploring biofertilizers have shown negative results there is significant evidence that these microorganisms can improve crop production factors and efficiency. There are indications that the consistency and benefits vary with crop and must be studied further.

While work has been accomplished to build a library of crop-biofertilizer compatibility, more research must be done to expand this library and address associated with the rhizosphere environment in hydroponics. Thus, the aim and objective of this study was to determine how common and commercially available microorganisms affect the growth, physiology, nutrient concentration, and nutrient uptake efficiency (NUE) of Black Summer Pac Choi (*Brassica rapa* var. chinensis) grown in Deep-Water System (DWS) and Nutrient Film Technique (NFT) hydroponics. We hypothesized that *Bacillus amyloliquefaciens* would enhance nutrient uptake, while *Trichoderma* spp. had more competitive interaction effects on Pac Choi grown in different hydroponic systems.

## 2 Materials and methods

### 2.1 Experimental conditions

This experiment was conducted in two separate trials in a controlled greenhouse environment at the Texas Tech University Horticulture Gardens and Greenhouse facility. Black Summer Pac Choi (*Brassica rapa* var. chinensis) was grown for five weeks in two seasons (September 2022 and March 2023) in NFT and DWS hydroponic systems. During plant growth, greenhouse temperature averaged 27.3°C in trial 1 and 29.6°C in trial 2. A temperature and humidity sensor (tempi.fi, Woburn, MA, USA) was suspended in the air between two levels of the NFT system, and a separate temperature and humidity sensor (HOBO, Bourne, MA, USA) was adjusted to leaf level in the DWS hydroponics system. The university greenhouse facilities preset the temperature, and readings were collected after each second in both trials and then averaged every hour.

### 2.2 Experimental setup

Each set up was conducted for two experimental trials. A DWS hydroponic system consisted of 12 seven L storage containers. Each container lid had six holes of 2 in diameter (5.08 cm) to accommodate net pots for plant placement (72 plants in total). Plastic tubing was connected to air pumps and fed through the lid to provide aeration to the roots. Supplemental lighting was provided by two LumiGrow TopLight Node light fixtures (42.6 in × 4.1 in) (LumiGrow, Emeryville, CA, USA) (λ: 400-700 nm) for six hours a day to achieve photosynthetically active radiation (PAR) between 405-407 µmol m^-2^ s^-1^ and an average DLI of 12 (mol m^-2^ day^-1^). DWS hydroponic containers were organized in a random block design with a treatment assigned randomly to each container. Three containers in total represented each treatment with a total of 18 sample replicates per treatment. Black Summer Pac Choi was also grown in an NFT hydroponic system that consisted of two towers with two levels per tower. Supplemental lighting was given for each level with KEGIAN light fixtures (23.6 in × 9.5 in)(Amazon, Seattle, WA, USA)(λ: 400-750 nm). Light PPFD output was adjusted at the bottom levels to reach approximately the same light intensity across all treatments and levels. After adjustments, average PAR and DLI were recorded as 405-407 µmol m^-2^ s^-1^ and 12 mol m^-2^ day^-1^, respectively. Each NFT tower level was assigned with an experimental treatment at random based on a random block design. Each treatment was assigned a matching sample replicate amount (18) as the DWS. Treatments described below were applied to the reservoirs of each system throughout the trials at the rates specified by the commercial manufacturer. The growth of a mycelial mat in all *Trichoderma* spp. treatment reservoirs during Trial 1 resulted in weekly reservoir changes. Trial 2 reservoirs were not completely drained and only filled when EC or water levels dipped below optimum. Both deep-water and NFT hydroponic systems received a 1:1 ratio of two liquid fertilizers Floragro (2-1-6) and Floramicro (5-0-1) (General Hydroponics, Santa Rosa, CA, USA) to maintain an EC (electrical conductivity) (Groline, Hanna Instruments, Smithfield, RI, USA) range of 1.5-2.5 ppm as well as received pH stabilizers to maintain a pH range of 5.5-7.5.

### 2.3 Microorganism treatments and inoculation

Experimental treatments consisted of three unique microorganism inoculations and a non-inoculated control. A commercial biofertilizer containing *Bacillus amyloliquefaciens* (Botanicare, Vancouver, WA, USA) was added directly to both DWS and NFT reservoirs at the inoculation rate of 2 mL/gal (1.0×104 CFU/mL). A separate commercial biofertilizer blend containing *Trichoderma harzianum* and *Trichoderma viride* (Mikrobs, Suwanee, GA, USA) was added directly to both DWS and NFT reservoirs at the inoculation rate of 2.5 g/gal (2.0x107 CFU/g). A third treatment consisted of a combination of *Bacillus amyloliquefaciens* and *Trichoderma* spp. commercial biofertilizers at an inoculation rate of 2 mL/gal and 2.5 g/gal, respectively. Inoculation rates were applied according to the biofertilizer manufacturer’s instructions. Both of the biofertilizers are known to associate with many plants and are endophytic in nature (Zalila-Kolsi et al., 2023). *Bacillus amyloliquefaciens* has also been associated with a wide variety of plants and is commonly found in the environment (Zalila-Kolsi et al., 2023). Further, *Trichoderma* has shown plant growth promotion effects on a wide variety of crops, due to production of auxin compounds and capacity for solubilizing nutrients like phosphate (Zin and Badaluddin, 2020; Tyśkiewicz et al., 2022). Research by Poveda and Eugui (2022) has also shown beneficial and synergistic effects on plants inoculated with both *Trichoderma* and *Bacillus* spp. Because both biofertilizers have been used on Pac choi successfully in other studies (Hirst et al., 2024) and are known to associate with many species, no compatibility tests were performed.

### 2.4 Physical analysis

#### 2.4.1 Growth index

Growth Index (GI) was calculated using Equation 1 according to the cylindrical shape of Pac Choi. GI measurements were collected and calculated every week of growth during Trial 2. GI was collected and calculated for all experimental replicates. Results were than plotted to show change in growth index over the period of five weeks.


(1) $$\frac{1}{2}W1*\frac{1}{2}W2*h*\pi$$

W1 = Width from north-south

W2 = Width from east-west

h = plant height from base of stem to tip of leaves

### 2.5 Chemical analysis

#### 2.5.1 Nutrient uptake efficiency

After the fifth week of growth, all replicate Black Summer Pac Choi leaf and stem samples were harvested, freeze-dried (Harvest Right, Salt Lake City, UT, USA) and shipped to Waters Agricultural Laboratories Inc. (Camilla, GA, USA) for a general nutrient analysis. All sample replicates were tested for concentrations of Nitrogen (N), Potassium (K), Phosphorus (P), Magnesium (Mg), Calcium (Ca), Sulfur (S), Boron (B), Zinc (Zn), Manganese (Mn), Iron (Fe), and Copper (Cu). After analysis, nutrient uptake efficiency (NUE) was calculated using Equation 2 according to (Dobermann, 2007)


(2) $$\left(\frac{gS}{gA}\right)*100$$

gS = grams NPK in shoots

gA = grams of NPK applied

### 2.6 Statistics

JMP 16.0.0 (SAS, Cary, NC, USA) software was used for standard least squares factorial analysis. Each trial performed was analyzed separately, and a single plant was considered one replication for each analysis. After each trial, 72 replicates were analyzed for physical and chemical analysis. Criteria for significant differences were set at p ≤ 0.05, and a student’s t-test further tested significant differences to determine mean separation.

## 3 Results

### 3.1 Physiology and growth

#### 3.1.1 Harvest results

In trial 1, the control and *Bacillus amyloliquefaciens* treatments resulted in significantly greater fresh shoot weight and root length in both systems (**Table 1**). However, when systems were analyzed separately, Pac Choi grown in NFT during trial 1 resulted in significantly higher fresh shoot weight in the control and Bacillus treatments, followed by the *Trichoderma* and combination treatments (p ≤ 0.0001; **Table 1**). Differences in root weight were seen between treatments, which yielded a significantly greater root weight in the *Trichoderma* spp. treatment in the first trial (p=0.0068). Root length was similar in both the DWS and NFT systems, where the control and *Bacillus* treatments had longer roots than those in the *Trichoderma* and combination treatments. This was further supported by the root:shoot analysis, which showed greater values in the *Trichoderma* containing treatments, particularly in the NFT system.

**Table 1:** Measurements of the fresh shoot and root weight (g) and root length (mm) of microorganism treatments during Trial 1 and Trial 2.

	Trial 1							
	DWS			NFT				
Treatments	Fresh Shoot Weight (g)	Fresh Root Weight (g)	Root Length (mm)	Root: Shoot	Fresh Shoot Weight (g)	Fresh Root Weight (g)	Root Length (mm)	Root: Shoot
Control	64.3^A^	39.8^BC^	496.8^A^	0.69	72.2^A^	18.9	499.6^A^	0.27^C^
*Bacillus amyloliquefaciens.*	57.5^A^	35.0^C^	604.0^A^	0.69	82.7^A^	16.5	577.3^A^	0.22^C^
*Trichoderma* spp.	28.0^B^	48.1^A^	166.1^B^	3.49	31.8^B^	18.0	307.5^B^	0.68^A^
Combination	25.9^B^	44.3^AB^	224.7^B^	2.11	46.1^C^	19.3	316.0^B^	0.50^B^
p values	*<0.0001*	*0.007*	*0.005*	0.084	*<0.0001*	0.364	*<0.0001*	*<0.0001*
	Trial 2							
	DWS			NFT				
Treatments	Fresh Shoot Weight (g)	Fresh Root Weight (g)	Root Length (mm)	Root: Shoot	Fresh Shoot Weight (g)	Fresh Root Weight (g)	Root Length (mm)	Root: Shoot
Control	85.9^A^	10.9^C^	40.0	0.15^B^	181.0^A^	34.5^B^	45.0^A^	0.34
*Bacillus amyloliquefaciens*	93.4^A^	15.4^B^	39.7	0.15^B^	148.7^AB^	32.9^B^	34.6^B^	0.25
*Trichoderma* spp.	36.3^B^	20.7^A^	52.3	0.83^A^	136.9^B^	38.3^AB^	33.7^B^	0.28
Combination	30.4^B^	15.2^BC^	46.6	0.85^A^	123.7^B^	41.7^A^	38.2^AB^	0.35
p values	*<0.0001*	*0.001*	0.688	*0.0005*	*0.019*	*0.015*	*0.024*	0.791

DWS, Deep Water System; NFT, Nutrient Film Technique.

Values followed by the same letter are not significantly different at the P<0.05 level.

p values were identified using a student’s t test.

In trial 2, pac choi grown in DWS culture yielded significantly greater fresh shoot weight in the control and *Bacillus amyloliquefaciens* treatments (p ≤ 0.0001; **Table 1**). Additionally, trial 2 Pac Choi grown in NFT yielded significantly greater fresh weight and root length in the control treatments (p=0.0190, p=0.0237, respectively). The root weight of Pac Choi grown in DWS was significantly greater in the *Trichoderma* spp., followed by the *Bacillus*, combination, and control treatments (**Table 1**). In the NFT system, the root weight of plants was significantly greater in the combination treatment, followed by the *Trichoderma* treatment; however, there was no significant difference between the control and *Bacillus* treatments (p=0.0010, p=0.0150). The root:shoot analysis also reflected similar findings with *Trichoderma* containing treatments having higher values, except in trial 2, these were only significant in the DWS units.

#### 3.1.2 Growth index

**Figure 1:**
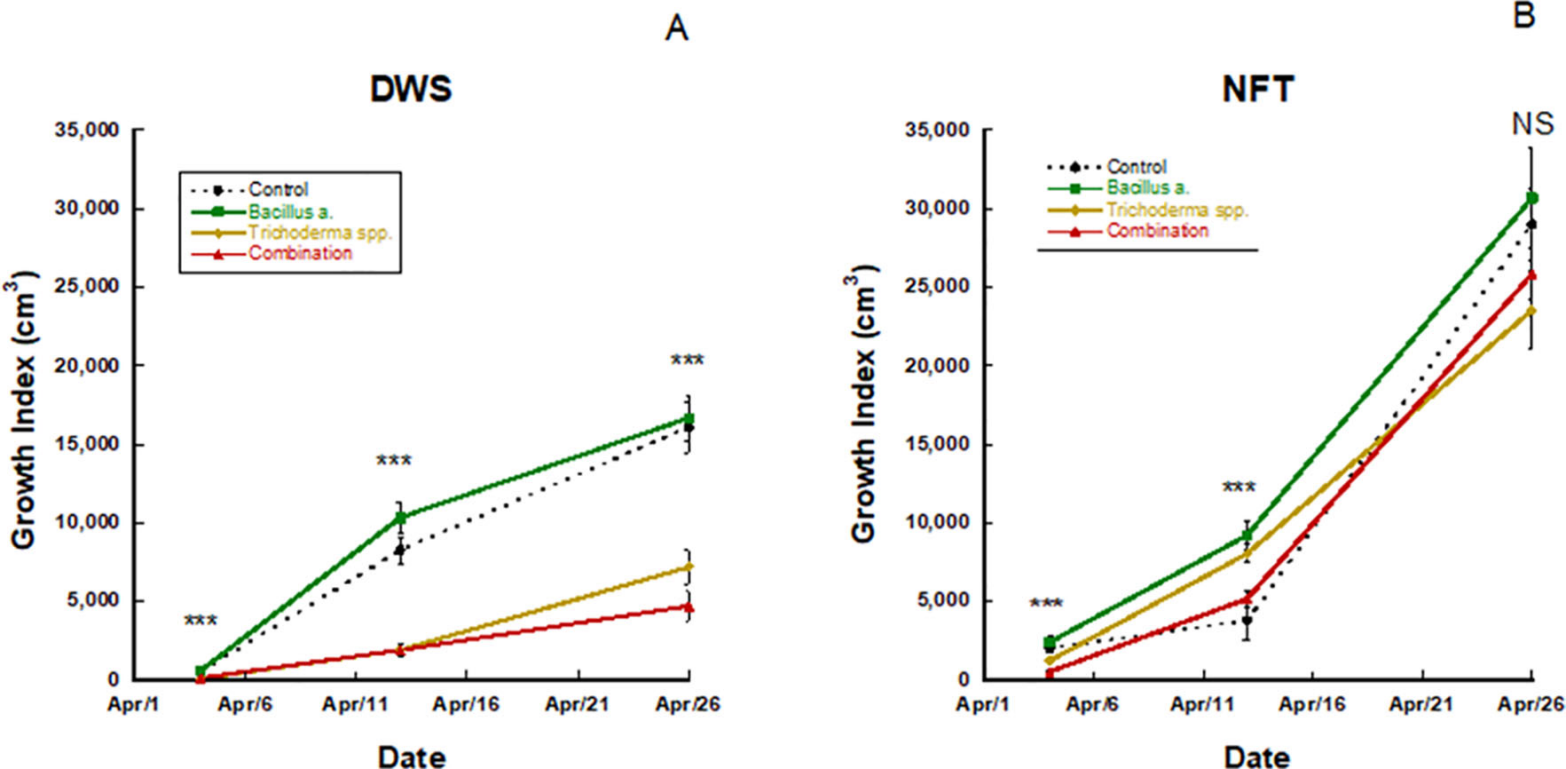
Pac choi growth index for **(A)** deep water (DWS) and **(B)** nutrient film technique (NFT) systems treated with different biofertilizers. The order of significance is represented by p<0.0001 (***). NS denotes no significant differences found. P-values were identified using a student's t-test. Bars represent ±1 standard error of the mean.

In the trial 2 DWS treatment, Pac Choi, in week 3 of growth, had a significantly higher growth index in the *Bacillus amyloliquefaciens* and control treatments (p ≤ 0.0001, p ≤ 0.0001, respectively; **Figure 1**), while the *Trichoderma* and combination treatments were statistically similar but lower than the other treatments. By the fourth week of growth, plants in the *Bacillus amyloliquefaciens* treatment had a greater growth index in the NFT culture only (p ≤ 0.0001). After the fifth and final weeks of growth, no significant differences existed between treatments for growth index in NFT culture (p=0.1812). However, in DWS culture, *Bacillus amyloliquefaciens* and control-treated Pac Choi had a significantly greater growth index, followed by *Trichoderma* and the combination treatment DWS (p ≤ 0.0001).

### 3.2 Plant nutrition

#### 3.2.1 Nutrient content

After the first trial, the control treatment yielded significantly greater concentrations of K in Pac Choi tissues in both hydroponic systems (**Table 2**). Furthermore, the control plants contained significantly greater Fe in the DWS-grown Pac Choi (p=0.023, **Table 2**). The control and *Bacillus amyloliquefaciens* treatments behaved similarly with increased Cu and P concentrations in Pac Choi grown in DWS (p=0.0004 for Cu, p=0.0003 for P, **Table 2**) as well as significantly greater N, Ca, and Zn concentrations in NFT grown Pac Choi (p<0.0001 for N, p=0.0006 for Ca, p=0.0093 for Zn, **Table 2**). For Pac Choi given the *Trichoderma* spp. treatment, plants contained significantly greater B concentrations in both systems (p=0.0006 for DWS, p ≤ 0.0001 for NFT, **Table 2**) but only significantly increased S concentrations in NFT (p=0.0002, **Table 2**). At the same time, the combination-treated plants contained significantly greater Mg concentrations in both systems (p=0.0006 for DWS, p=0.0008 for NFT, **Table 2**). Finally, DWS-grown Pac Choi contained significantly greater nutrient concentrations for all nutrients significantly affected by treatment than NFT-grown Pac Choi.

**Table 2:** Nutrient concentrations of Pac Choi in different treatments and production systems for Trial 1 and Trial 2.

Trial 1											
Deep-Water Culture											
Treatments	N (%)	P (%)	K (%)	Ca (%)	Mg (%)	S (%)	B (ppm)	Zn (ppm)	Mn (ppm)	Fe (ppm)	Cu (ppm)
Control	7.52	0.97^A^	7.65^A^	3.93	0.24^C^	1.14	46.8^B^	31.0	177.8^A^	136.4	12.6^A^
*Bacillus amyloliquefaciens*	7.44	0.90^AB^	7.63^A^	3.55	0.23^C^	1.19	46.8^B^	32.0	162.6^A^	103.8^B^	11.6^A^
*Trichoderma* spp.	7.18	0.82^B^	6.78^AB^	3.77	0.28^B^	1.27	50.2^B^	32.0	161.4^A^	95.8^B^	8.2^B^
Combination	7.04	0.71^C^	6.35^B^	3.58	0.33^A^	1.25	67.2^A^	33.8	130.8^B^	97.6^B^	8.0^B^
p values	0.072	*0.0003*	*0.018*	0.146	*0.0006*	0.099	*0.0006*	0.826	*0.021*	*0.023*	*0.0004*
NFT											
Treatments	N (%)	P (%)	K (%)	Ca (%)	Mg (%)	S (%)	B (ppm)	Zn (ppm)	Mn (ppm)	Fe (ppm)	Cu (ppm)
Control	7.07^A^	0.80	7.06^A^	2.87^AB^	0.21	0.91^B^	44.2^B^	23.8^AB^	195.8^A^	92.4	4.00
*Bacillus amyloliquefaciens*	7.25^A^	0.90	6.50^B^	2.53^B^	0.19	0.94^B^	42.8^B^	26.6^A^	196.6^A^	91.2	5.20
*Trichoderma* spp.	6.39^C^	0.80	5.95^C^	2.12^C^	0.23	1.11^A^	54.2^A^	23.0^BC^	171.4^B^	86.8	5.40
Combination	6.75^B^	0.87	6.35^BC^	2.95^A^	0.25	1.09^A^	58.4^A^	22.0^C^	147.0^C^	94.4	5.00
p values	*<.0001*	0.097	*0.004*	*0.0006*	0.050	*<.0001*	*<.0001*	*0.009*	*0.001*	0.753	0.057
Trial 2											
Deep-Water Culture											
Treatments	N (%)	P (%)	K (%)	Ca (%)	Mg (%)	S (%)	B (ppm)	Zn (ppm)	Mn (ppm)	Fe (ppm)	Cu (ppm)
Control	5.73^A^	0.66^A^	5.89	3.69^A^	0.22^C^	1.08^B^	42.5^BC^	42.5	222.3^A^	161.5	20.0^A^
*Bacillus amyloliquefaciens*	5.62^A^	0.52^B^	5.78	3.69^A^	0.24^B^	1.13^B^	40.6^C^	30.6	132.2^C^	129.2	6.2^B^
*Trichoderma* spp.	5.32^B^	0.41^C^	5.62	2.80^C^	0.25^AB^	1.29^A^	49.2^B^	29.2	170.4^BC^	118.2	7.0^B^
Combination	5.78^A^	0.54^B^	5.35	3.23^B^	0.27^A^	1.35^A^	57.4^A^	34.4	203.2^AB^	162.4	11.4^AB^
p values	*0.007*	*<.0001*	0.108	*<.0001*	*0.0008*	*0.0002*	*0.0005*	0.093	*0.004*	0.135	*0.020*
NFT											
Treatments	N (%)	P (%)	K (%)	Ca (%)	Mg (%)	S (%)	B (ppm)	Zn (ppm)	Mn (ppm)	Fe (ppm)	Cu (ppm)
Control	5.45^C^	0.53^B^	5.09^AB^	3.24	0.21^C^	0.81^B^	47.0^B^	27.4^C^	226.8^A^	139.2	5.40
*Bacillus amyloliquefaciens*	6.08^A^	0.67^A^	5.21^A^	2.96	0.38^A^	1.06^A^	59.8^A^	34.4^A^	175.8^B^	135.2	6.60
*Trichoderma* spp.	5.23^C^	0.50^B^	4.66^B^	3.05	0.26^B^	0.72^C^	59.2^A^	28.2^BC^	195.2^B^	144.2	6.20
Combination	5.76^B^	0.65^A^	5.26^A^	2.99	0.36^A^	0.97^A^	55.6^A^	30.6^B^	149.6^C^	136.0	5.80
p values	*<.0001*	*<.0001*	*0.049*	0.154	*<.0001*	*<.0001*	*<.0001*	*0.0005*	*<.0001*	0.298	0.136

Values followed by the same letter are not significantly different at the P<0.05 level.

p values were identified using a student’s t-tests.

After the second trial, the control treatment yielded Pac Choi with significantly greater concentrations of Mn in both systems (**Table 2**). Additionally, trial 2 DWS Pac Choi had greater concentrations of P and Cu when given the control treatment (p ≤ 0.0001 for P, p=0.02 for Cu, **Table 2**) but had significantly greater B concentrations in NFT (p ≤ 0.0001, **Table 2**). The *Bacillus amyloliquefaciens* and combination treatments showed higher Mg levels in plants grown in both systems during trial 2 (p=0.004 for DWS, p ≤ 0.0001 for NFT, **Table 2**), but the two treatments also showed higher P and K in the NFT culture only (p=0.0001 for P, p=0.049 for K, **Table 2**). Pac Choi grown with the combination treatment contained significantly greater Ca and B concentrations in DWS culture only (p=0.0006 for Ca, p=0.0005 for B, **Table 2**).

#### 3.2.2 Nutrient uptake efficiency

During the first trial, Pac Choi grown with the control treatment had the greatest P and K uptake efficiency in both systems (p=0.0013, **Table 3**). However, the *Bacillus amyloliquefaciens* treated plants did not significantly differ in P and K nutrient uptake efficiency in the DWS. However, Pac Choi grown under the control and *Bacillus amyloliquefaciens* treatments contained significantly greater N uptake efficiency in NFT culture only (p=0.004, **Table 3**). In trial 1, lower nutrient uptake efficiencies were generally seen in the combination or *Trichoderma* spp. treatments. In trial 2, Pac Choi grown with *Bacillus amyloliquefaciens* and the combination treatment contained significantly greater N uptake efficiency in DWS (p=0.0002, **Table 3**). *Bacillus amyloliquefaciens* containing cultures yielded plants with greater K uptake efficiency (p=0.0003, **Table 3**) but were not significantly greater than the combination treatments. The combination treatment yielded significantly greater P uptake efficiency (p=0.0058, **Table 3**) followed by the control, *Bacillus amyloliquefaciens*., and *Trichoderma* spp. treatments. The NFT system had no significant differences between treatments for any nutrient uptake efficiencies in trial 2.

**Table 3:** Nitrogen (N), Phosphorous (P), and Potassium (K) Nutrient Uptake Efficiency (NUE) (%) of microorganism treatments during Trial 1 and Trial 2.

	Trial 1					
	DWS				NFT		
Treatments	N NUE (%)	P NUE (%)	K NUE (%)	N NUE (%)	P NUE (%)	K NUE (%)
Control	3.27	4.20^A^	4.75^A^	1.21^A^	1.38	1.73^A^
*Bacillus amyloliquefaciens*	3.38	4.11^AB^	4.98^A^	1.21^A^	1.51	1.55^B^
*Trichoderma* spp.	3.27	3.73^B^	4.41^AB^	1.10^C^	1.37	1.46^B^
Combination	3.20	3.25^C^	4.13^B^	1.56^B^	1.49	1.56^B^
p values	0.240	*0.001*	*0.037*	*<.0001*	0.212	*0.004*
	Trial 2					
	DWS			NFT		
Treatments	N NUE (%)	P NUE (%)	K NUE (%)	N NUE (%)	P NUE (%)	K NUE (%)
Control	3.88^B^	4.43^AB^	5.70^B^	2.31	2.26	3.07
*Bacillus amyloliquefaciens.*	6.53^A^	3.23^BC^	9.67^A^	1.93	2.10	2.36
*Trichoderma* spp.	2.01^C^	1.57^C^	3.02^C^	2.05	1.94	2.58
Combination	5.82^A^	5.46^A^	7.62^AB^	1.99	2.24	2.58
p values	*0.0002*	*0.006*	*0.0003*	0.591	0.717	0.324

Deep water culture (DWS) and nutrient film technique system (NFT).

Values followed by the same letter are not significantly different at the P<0.05 level.

p values were identified using a student’s t-test

## 4 Discussion

Interest in biofertilizers and beneficial fungi has grown in recent years. They have been widely shown to improve plant yields and performance through associations with different crops. These biofertilizers are an essential element to sustainable horticulture and urban agriculture, whether the intended results are increased yield, decreased plant pathogens, or both (Gül et al., 2008; Kıdoğlu et al., 2009; Lee and Lee, 2015). For example, Dasgan et al. (2022) found that Basil (*Ocimum basilicum* L. ‘Dino’) had increased N, P, K, Mg, and Fe concentrations along with yield and leaf area when given bacterial, mycorrhizal, and micro-algae biofertilizers in a hydroponic setting. Tyśkiewicz et al. (2022) also explored the current uses and limitations of *Trichoderma* spp. as a biocontrol agent for plant pathogens and the properties associated with plant growth promotion. Further, Reed and Glick (2023) discussed the variable relationship between biofertilizers and vegetable crops while Backer et al. (2018) discussed how the root environment can alter compatibility and behavior. This discrepancy between biofertilizer and plant associations has not been exhaustively studied due to the wide variety of plants and biofertilizers available. Furthermore, there are limited studies on their impacts and efficacy in hydroponic production systems. To achieve optimal beneficial effects from biofertilizers, compatibility between microorganisms and plants and the rhizosphere environment must be studied.

### 4.1 Growth promotion

Biofertilizers are added to the soil environment of vegetable crops for their plant growth-promoting properties. For example, adding a *Bacillus amyloliquefaciens* strain increased tomatoes’ fresh shoot and root growth (Samaras et al., 2021). Furthermore, the growth-promoting properties of *Bacillus amyloliquefaciens* have been recorded in tea plants, where bud weight was increased by 22% in plants that had been inoculated (Bai et al., 2014). Results from our study show similar impacts of *Bacillus amyloliquefaciens* on hydroponically grown Black Summer Pac Choi, where growth-promoting properties were seen in fresh weight, root length, and growth index. However, these results vary with the microorganism and crop studied. Interestingly, there were few interaction effects of production system and biofertilizer treatment on plant growth factors. This could indicate that patterns may be independent of systems producing the plants or that the effects of the treatments are similar in each system.

In this study, *Trichoderma* spp. negatively impacted fresh weight, root length, and growth index of Pac Choi. This contrasts with other experiments where *Trichoderma* biofertilizers increased plant growth. These conflicting results may be due to the difference in production systems used. *Trichoderma* has shown primarily beneficial impacts in soil-based studies, while there were few if any benefits in hydroponic systems. For example, Bader et al. (2020) demonstrated that *Trichoderma harzianum* promoted soil-grown tomato (*Solanum lycopersicum* L.) shoot growth and increased the fresh weight of both shoots and roots. In addition, Dabire et al. (2016) concluded that soil-grown onion root tissues, fresh weight, and the number of leaves increased up to 30 days after inoculating seeds and seedling beds with a native strand of *Trichoderma harzianum*. In a hydroponic system, Moreira et al. (2022) also showed that lettuce yield was increased after applying *Trichoderma harzianum* to a reservoir with reduced EC. Similarly, cucumbers grown in a gnotobiotic hydroponic system experienced as much as a 40% increase in dry weight of shoots after inoculation with *Trichoderma harzianum* (Yedidia et al., 2001). However, our data conflict with these findings and show that applying multiple *Trichoderma* spp. reduced Black Summer Pac Choi growth. This could have been a function of species found in the commercial blend, application rates, and/or other factors that affect the growth of *Trichoderma* in the hydroponic systems. The presence of a mycelial mat attached to the root system of both DWS and NFT could also explain this behavior. The closed-loop recirculation of nutrient solutions in the hydroponics system is likely to encourage the growth of *Trichoderma* spp., potentially leading to larger colonies that could compete with plants for nutrients. As *Trichoderma* is a very aggressive and opportunistic fungus (Schuster and Schmoll, 2010), the large population likely outcompeted the Black Summer Pac Choi for nutrients and oxygen. Large colonies were observed to surround and become attached to the roots of plants in both the NFT and DWS hydroponics. This observation is further quantified in **Table 1** where fresh root weight was significantly greater in treatments containing *Trichoderma.* This could also explain why plants, given this biofertilizer, grew smaller overall. This could possibly be mitigated by using lower inoculation rates, because those provided by the manufacturer may not be conducive for hydroponic production. However, future research should expand on this to determine what factors affect *Trichoderma* colonization in hydroponic systems.

### 4.2 Impacts on nutrition of Black Summer Bac Choi

After a complete nutrient evaluation, the control and *Bacillus amyloliquefaciens* treatments behaved similarly with few exceptions. According to the results shown in **Table 2**, *Bacillus amyloliquefaciens* had the potential to facilitate the increase concentrations of certain nutrients (N, P, K, Cu, Zn, Mg) in DWS and NFT hydroponics but may not perform better than a system without biofertilizers. This information differs from other sources that claim *Bacillus* spp. increased plant nutrition in both soil systems and hydroponics (Luo et al., 2022; Oliveira et al., 2023; Sharma et al., 2013; Wang et al., 2022). However, Luo et al. (2022); Sharma et al. (2013), and Wang et al. (2022) all argue that the mechanisms for increasing shoot nutrition occur through increasing nutrient availability during environmental stress. One reason for the similarity between the control treatment and the *Bacillus amyloliquefaciens* treatment could be that there was already an abundance of bioavailable nutrients before the biofertilizer was applied, and thus no additional benefits could be achieved. As a second argument, the inoculation rates or compatibility between *Bacillus amyloliquefaciens* would show similar results to those seen in this study. However, evidence suggests compatibility between *Bacillus* spp. and Chinese cabbage. Ku et al. (2018) demonstrated that field inoculations of *Bacillus cereus* resulted in an increased yield of Chinese cabbage. Kang et al. (2019) also demonstrated that soil inoculation of *Bacillus subtilis* increased nutrient uptake in Chinese cabbage. Nevertheless, inadequate inoculations would also provide evidence for the results demonstrated in this study and the use of inorganic nutrients instead of organic nutrients. Inorganic nutrients are often used in hydroponics as they allow for better pH control, easily adjustable rates for plant growth stage, and limit phytotoxic organic compounds (Torres and Somera, 2023). However, the commercial blend recommended pairing the inoculation with organic nutrients as the biofertilizer aided in providing bioavailable nutrients.

On the other hand, *Trichoderma* spp. resulted in negative impacts on most nutrient concentrations in the Black Summer Pac Choi shoot tissue. While there was no sign of any nutrient insufficiency (Uchida, 2000), concentrations were significantly reduced compared to the control treatment. Even when combined with *Bacillus amyloliquefaciens*, only B, S, and Mg concentrations were higher than control. Moreira et al. (2022) document similar results where inoculation of *Trichoderma harzianum* in hydroponically grown lettuce resulted in no effects on nutrient concentration. The results continue to indicate that *Trichoderma* is likely competing with the Pac Choi plants for nutrients in the nutrient solutions, as discussed in the previous section. Since fewer nutrients were taken into the shoots and roots, less growth could occur in the Pac Choi. The benefits of *Trichoderma* spp. seen in soil systems may be due to more soil microorganisms that compete or the natural soil structure that allows for more beneficial associations with plant roots (Chewapanich et al., 2021; Giurgiu et al., 2018; Promwee and Intana, 2022).

Black Summer Pac Choi treated with *Bacillus amyloliquefaciens* did not have a significantly greater NUE than the control in either trial of DWS and NFT. One suggestion is that there was no aid in nutrient uptake from *Bacillus amyloliquefaciens* This is a direct contradiction to de Sousa et al. (2021) and Wang et al. (2023), where nutrient uptake was increased by *Bacillus* spp. in both soil and hydroponic systems. However, these results match nutrient concentration results. The lack of significant effects of *Bacillus amyloliquefaciens* on nutrient concentrations in Pac Choi could be due to the abundance of bioavailable nutrients already possessed in the fertilizer additions. Since there were no nutrient deficiencies, *Bacillus amyloliquefaciens* likely had little effect on nutrient uptake. Mourouzidou et al. (2023) support this process and present that *Bacillus* spp. can increase nutrient uptake during N and Fe deficiencies and saline environments. This is also supported by the manufacturer’s recommendations for use with organic fertilizers, as discussed previously. Alternatively, the potential compatibility, or lack thereof, could explain these effects between species. If Black Summer Pac Choi is incompatible with *Bacillus amyloliquefaciens*, similar results would be shown between the control treatment and *Bacillus amyloliquefaciens* treatment in nutrient concentration and NUE.

Nutrient uptake efficiency varied with treatments, and while Trichoderma-containing treatments were, on average, lower in NUE than others, these findings were inconsistent between trials and systems. Additionally, N NUE and K NUE showed significant interaction effects between treatments and systems. This showed that the treatments did have different effects in different systems, but in general the deep water grown plants had higher NUE. Overall, there was a reduction of NUE occurring in both DWS and NFT for N, P, and K treated with *Trichoderma*. Both physiology and nutrient concentration results have suggested that the presence of *Trichoderma* spp. at the inoculation rate likely outcompeted Black Summer Pac Choi for nutrients. Moreover, results from the NUE analysis indicate that the *Trichoderma* fungus negatively impacted the ability of the plant to take up nutrients due to competition. At the inoculation rates used, *Trichoderma* spp. is not a beneficial biofertilizer for Black Summer Pac Choi grown in DWS and NFT hydroponics. In their study, Li et al. (2015) discusses both patterns of symbiosis between *Trichoderma* spp. and soil-grown tomatoes. Under Cu-deficient conditions, *Trichoderma* spp. increased the Cu intake of tomato seedlings by 42%. However, under P and Zn-deficient conditions, *Trichoderma* spp. was shown to compete with and negatively impact the P and Zn concentrations of tomato seedlings. This suggests that more research must examine fertilizer composition, inoculation rates and population sizes of *Trichoderma* spp. in hydroponics.

Commercial hydroponic production focuses on efficient and rapid production of crops, primarily leafy green vegetables. One of the primary issues with hydroponic production is that the use of organic fertilizers is not recommended because of the necessity for microorganisms to mineralize nutrients for absorption (McClintic et al., 2024). The use of microorganisms and biofertilizers that aid in this process is necessary to facilitate this but can offer other benefits as previously discussed. However, the practical application of commercial biofertilizers must be examined in different crops and production systems. This research demonstrates that manufacturers recommended rates may not be practical for use in hydroponic production due to mycelial mat formation and the proliferation of Trichoderma supplied with adequate nutrients. These studies replicated greenhouse grower conditions and, as such, showed that the results often promised are not always delivered. Consequently, future research should investigate different inoculation rates of biofertilizers, the mechanisms of the interactions between plants and biofertilizers, and different formulations or ratios of the biofertilizers or organisms.

## 5 Conclusion

The use of *Bacillus amyloliquefaciens* as a biofertilizer for Black Summer Pac Choi in a hydroponic environment has the potential for positive effects, especially for an early harvest. The effects of *Bacillus* were primarily seen on plant physiology and nutrient uptake but weren’t significantly different from the control in most cases. However, the use of *Trichoderma* spp. at the tested inoculation rate is not recommended. *Trichoderma* negatively affected yield and aerial tissue factors, and while root data indicate that roots were larger, it was due to mycelial mats that accumulated. While both production systems showed positive impacts on growth, NFT grown plants were generally larger.Future research should aim to refine inoculation methods and rates for biofertilizers and test additional biofertilizer types for Black Summer Pac Choi. Further research must be completed to evaluate the differences in biofertilizer behavior in organically and inorganically fertilized systems.

## Data Availability

The raw data supporting the conclusions of this article will be made available by the authors, without undue reservation.
