# Supplementary figures and images for: Impacts of Bacillus amyloliquefaciens and Trichoderma spp. on Pac Choi (Brassica rapa var. chinensis) grown in different hydroponic systems
Source: Front Plant Sci. 2024 Sep 23;15:1438038. doi: 10.3389/fpls.2024.1438038 (PMC11456494; doi:10.3389/fpls.2024.1438038)

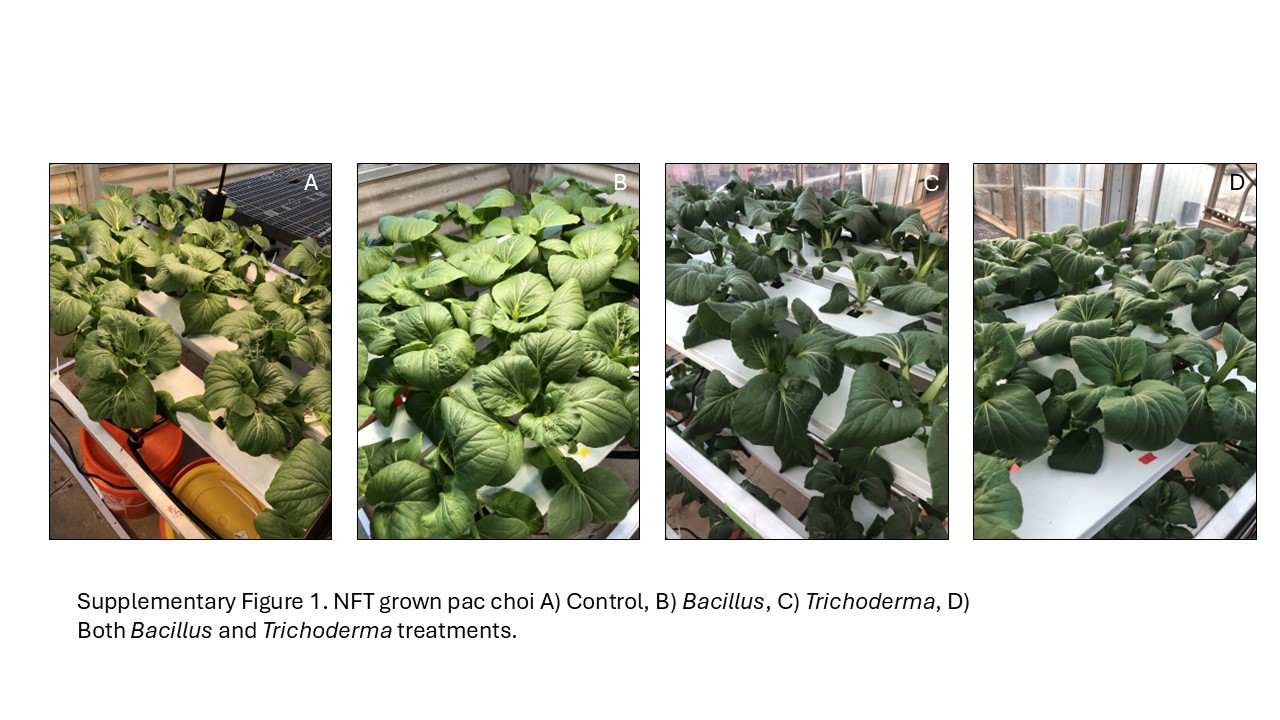

Supplement: Supplementary file 1 [file Image1.jpeg]
